# Clusterin ameliorates tau pathology in vivo by inhibiting fibril formation

**DOI:** 10.1186/s40478-020-01079-1

**Published:** 2020-12-01

**Authors:** Aleksandra M. Wojtas, Yari Carlomagno, Jonathon P. Sens, Silvia S. Kang, Tanner D. Jensen, Aishe Kurti, Kelsey E. Baker, Taylor J. Berry, Virginia R. Phillips, Monica Casey Castanedes, Ayesha Awan, Michael DeTure, Cristhoper H. Fernandez De Castro, Ariston L. Librero, Mei Yue, Lillian Daughrity, Karen R. Jansen-West, Casey N. Cook, Dennis W. Dickson, Leonard Petrucelli, John D. Fryer

**Affiliations:** 1 Department of Neuroscience, Mayo Clinic, Collaborative Research Building CR03-010 13400 E. Shea Blvd, Scottsdale, AZ 85259 USA; 2 Department of Neuroscience, Mayo Clinic, Jacksonville, FL 32224 USA; 3 Neuroscience Graduate Program, Mayo Clinic Graduate School of Biomedical Sciences, Scottsdale, AZ 85259 USA

**Keywords:** Clusterin, Alzheimer’s disease, Tauopathy, Tau

## Abstract

The molecular chaperone Clusterin (CLU) impacts the amyloid pathway in Alzheimer’s disease (AD) but its role in tau pathology is unknown. We observed CLU co-localization with tau aggregates in AD and primary tauopathies and CLU levels were upregulated in response to tau accumulation. To further elucidate the effect of CLU on tau pathology, we utilized a gene delivery approach in CLU knock-out (CLU KO) mice to drive expression of tau bearing the P301L mutation. We found that loss of CLU was associated with exacerbated tau pathology and anxiety-like behaviors in our mouse model of tauopathy. Additionally, we found that CLU dramatically inhibited tau fibrilization using an in vitro assay. Together, these results demonstrate that CLU plays a major role in both amyloid and tau pathologies in AD.

**Electronic supplementary material:**

The online version of this article (10.1186/s40478-020-01079-1) contains supplementary material, which is available to authorized users.

## Introduction

Alzheimer’s disease (AD) is a highly prevalent neurodegenerative disorder characterized by deposition of amyloid-β (Aβ) peptide as parenchymal plaques [7] and aggregation of hyperphosphorylated protein tau as neurofibrillary tangles (NFTs) [8]. Aberrant tau accumulation is also a pathological hallmark of other neurodegenerative disorders, collectively known as primary tauopathies, including progressive supranuclear palsy (PSP), corticobasal degeneration (CBD), Pick’s disease (PiD), and frontotemporal dementia with parkinsonisms linked to chromosome 17 (FTDP-17), in which amyloid pathology is not usually observed [23]. Human tauopathies are often classified based on the presence of different tau isoforms that contain three or four carboxy-terminal repeat domains in the tau aggregates, including 3R (PiD), 4R (PSP, CBD), and 3R + 4R (AD) [12, 23]. Given that tau accumulation strongly correlates with neuronal dysfunction and cognitive impairment in AD and other tauopathies [1, 11], identifying factors that regulate tau aggregation in the brain is critical.

Compelling evidence implicates the clusterin (*CLU*) gene in AD pathophysiology. Several large-scale genome-wide association studies (GWAS) have identified a number of single nucleotide polymorphisms (SNPs) at the *CLU* locus that are significantly associated with altered AD risk [9, 13]. In addition, levels of CLU (also known as apolipoprotein J, apoJ) have been found to be increased in AD-affected brain regions [15] and CLU is highly elevated in the cerebrospinal fluid [16] and plasma of AD patients [19]. Previous studies have shown that CLU binds to Aβ affecting its deposition [4, 24], fibrillogenesis [17], and clearance [2, 27], thus suggesting that CLU is a key regulator of Aβ metabolism. Moreover, CLU involvement in a plethora of physiological and pathological processes have been attributed to its prominent role as a major extracellular chaperone [5]. However, it is unknown whether CLU affects AD risk by modulating tau pathology independently of Aβ.

## Materials and methods

### Animals

6-Month-old C57BL/6J (CLU wild-type, CLU WT) and CLU knock-out (CLU KO) mice were used in this study. Mice were housed in a temperature and humidity-controlled environment under a 12-h light/dark cycle and with free access to food and water. All studies were performed in accordance with *National Institute of Health Guide for the Care and Use of Laboratory Animals (*National Research Council (2011) Guide for the Care and Use of Laboratory Animals (National Academies Press, Washington, DC), 8th Ed.) under the approved protocol from the Mayo Clinic Institutional Animal Case and Use Committee. Both male and female mice were included in the study.

### Histological and biochemical evaluation of human postmortem tissue

To examine CLU association with tau, human postmortem brain tissues were obtained from Mayo Clinic Jacksonville Brain Bank from age-matched individuals diagnosed with primary and secondary tauopathies, including CBD, PiD, AD, and non-demented controls (Additional file 1: Table S1). For histological analyses, paraffin-embedded hippocampal sections were cut 5 μm, then deparaffinized using xylene and rehydrated in a graded series of alcohols. Antigen retrieval was achieved by incubating tissue sections in distilled water for 30 min under high temperature. Immunostaining was performed on the Thermo Scientific Autostainer 480S, using the Envision G/2 doublestain kit (Dako, Carpinteria, CA). Peroxidase (HRP) labeling of rabbit polyclonal Clusterin antibody (1:2500, Abcam, Cambridge, MA) was visualized using 3, 3′-diaminobenzidine (DAB +) as the chromogen. Alkaline phosphatase labeling of mouse monoclonal antibody MC1 (1:250, gift from Peter Davies, Feinstein Institute for Medical Research), was visualized using Vector Blue Alkaline Phosphatase Substrate kit (Vector Laboratories, Burlingame, CA). The sections were then dehydrated, coverslipped without counterstaining using permanent mounting media, scanned using the Aperio Slide Scanner (Aperio, Vista, CA), and analyzed using the ImageScope software (Aperio, Vista, CA).

Human brain sections were also incubated overnight with goat anti-CLU antibody (1:50, sc-6420, Santa Cruz Biotechnology) followed by double labeling with thioflavine-S to detect mature tau tangles. The slides were then incubated with Sudan black B (Sigma S0395) for 30 min at RT in order to eliminate lipofuscin-like autofluorescence. The images were captured using a Zeiss LSM 880 confocal microscope.

For biochemical analysis, frontal cortex from tauopathy cases and non-demented individuals was used. Demographic and neuropathological features of human subjects are presented in Additional file 1: Table S2. TBS buffer (50 mM Tris, pH8.0, 274 mM NaCl, 5 mM KCl, 1 mM PMSF, and protease and phosphatase inhibitor cocktail) was added to cortex and manually homogenized using glass dounce homogenizator. The homogenates were then ultracentrifuged at 150,000×*g* for 15 min at 4 °C and the supernatant (S1 fraction) was transferred to a new Eppendorf tube. The total CLU levels in the S1 fraction from human tissues were measured using the Human Clusterin DuoSet enzyme-linked immunosorbent assay (ELISA) (R&D Systems, DY5874) according to the manufacturer’s instructions. Total protein concentrations used for normalization were assessed by Bicinchoninic Acid (BCA) Protein Assay kit (Thermo Scientific), according to the manufacturer’s instructions with a standard curve using BSA.

### AAV-GFP and AAV-Tau^P301L^ viral production

Viral vector construction and AAV9 production was performed, as previously described [3]. Briefly, V5-tagged Tau^P301L^ or GFP expression plasmids were cloned into an AAV vector. The constructs were sequence-verified using ABI3730 with Big Dye chemistry (Applied Biosystems, Foster City, CA). AAV vectors expressing GFP and Tau^P301L^ under the control of the cytomegalovirus enhancer/chicken β-actin promoter, as well as a woodchuck post-transcriptional regulatory element and the bovine growth hormone polyA, were co-transfected with AAV helper plasmids into HEK293T cells. Cells were harvested and lysed in the presence of 0.5% sodium deoxycholate and 50 U/ml Benzonase (Sigma, St. Louis, MO) by freeze thawing 48 h post-transfection, and the virus was isolated using a discontinuous iodixanol gradient. Quantitative PCR was used to measure the genomic titer of each virus.

### Intracerebroventricular injections

AAV9-GFP or AAV9-Tau^P301L^ viruses were injected bilaterally into cerebral lateral ventricles of C57BL/6 J (CLU WT) and CLU KO pups at postnatal day 0 with 2E + 10 viral particles/ventricle. Briefly, newborn pups were cryoanasthesized and placed on a cold metal plate followed by piercing the skull with the 30-gauge needle just posterior to Bregman and 2 mm lateral to the midline. 2 μl of AAV virus was injected into the each lateral ventricle. Following the injections, pups were placed on the warm pad until they regained normal color and resumed movement.

### Behavioral assessment

The behavioral analysis consisting of open field test (OFA), elevated plus maze (EPM), and contextual and cued fear conditioning (CFC) was performed on 6-month-old CLU WT and CLU KO mice injected with AAV-GFP or AAV-Tau^P301L^. Mice were acclimated to the testing room for 1 h. All behavioral equipment was extensively cleaned with 30% ethanol between each animal. After each test animals were returned to their home cages. In the *OFA* *test,* that is performed to assess locomotor activity and anxiety, mice were placed in the center of the open-field arena (40 × 40 × 30 cm, *W* × *L* × *H*) and allowed to roam freely for 15 min. An overhead camera was used to track movement with AnyMaze software (Stoelting Co., Wood Dale, IL), and mice were analyzed for multiple measures, including total distance traveled, average speed, time mobile, and distance traveled in an imaginary ‘center’ zone (14 × 14 cm). The *EPM* test is conducted to test for anxiety and the entire maze is elevated 50 cm from the floor and consists of four arms (50 × 10 cm) with two of the arms enclosed with roofless gray walls (35 × 15 cm, *L* × *H*). Mice were tested by placing them in the center of the maze facing an open arm, and their behavior was tracked for 5 min with an overhead camera and AnyMaze software. The *CFC* test, which is used to assess hippocampal and amygdala associative learning and memory, was conducted in a sound attenuating chamber with a grid floor capable of delivering an electric shock, and freezing was measured with an overhead camera and FreezeFrame software (Actimetrics, Wilmette, IL). Mice were initially placed into the chamber undisturbed for 2 min, during which time baseline freezing behavior was recorded. An 80-dB white noise served as the conditioned stimulus (CS) and was presented for 30 s. During the final 2 s of this noise, mice received a mild foot shock (0.5 mA), which served as the unconditioned stimulus (US). After 1 min, another CS-US pair was presented. The mice were removed 30 s after the second CS-US pair and returned to its home cage. Twenty-four hours later, each mouse was returned to the test chamber and freezing behavior was recorded for 5 min (context test). Mice were returned to their home cage and placed in a different room than previously tested in reduced lighting conditions for a period of no less than 1 h. For the auditory CS test, environmental and contextual cues were changed, as previously described [3]. The animals were placed in the apparatus for 3 min and the auditory CS was presented and freezing was recorded for another 3 min (cued test). Baseline freezing behavior obtained during training was subtracted from the context or cued test to control for animal variability.

### Histological analyses

Following behavioral tests, 6-month-old mice were deeply anesthetized with pentobarbital (100 mg/kg i.p.) and transcardially perfused with phosphate buffered saline (PBS) to remove blood from cerebrovasculature. After brain removal, one hemibrain was drop-fixed in 10% neutral buffered formalin (Fisher Scientific, Waltham, MA) overnight at 4 °C followed by switching the brain to PBS and stored at 4 °C until histological evaluation. Cortex and hippocampus from the other hemibrain were isolated, frozen on dry ice and stored at − 80 °C until further processing. Histology was performed on the formalin-fixed hemibrains from CLU WT and CLU KO animals injected with AAV-GFP or AAV-Tau^P301L^ that were embedded in paraffin, sectioned in a sagittal plane at a thickness of 5 μm, and mounted on a glass slide. The sections were deparaffinized, rehydrated, and subjected to antigen-retrieval, followed by blocking endogenous peroxidase activity using 0.03% hydrogen peroxide for 30 min at RT. To investigate CLU association with tau deposits brain tissues from CLU WT animals injected with AAV-GFP or AAV-Tau^P301L^ were incubated overnight with goat anti-CLU antibody (1:50, sc-6420 Santa Cruz Biotechnology) and double labeled with thioflavine-S. Images were obtained on a Zeiss LSM 880 confocal microscope. For immunohistochemistry, brain sections were immunostained using the DAKO Autostainer (DAKO North America, Carpinteria, CA) and the DAKO EnVision + HRP system. The CP13 (pS202, 1:1000), PHF-1 (pS396/S404, 1:1500), and MC-1 (conformational epitope, 1:500) antibodies, kindly provided by Dr. Peter Davies (Feinstein Institute for Medical Research, North Shore LIJ Health Care System), and the anti-GFAP (1:2500 Cell Signaling Technology, 3670 Danvers, MA) and anti-IBA1 (1:3000, Wako Chemicals, 019-19741, Richmond, VA) antibodies were used in the study. Gallyas silver stain was performed on a subset of CLU WT and CLU KO animals injected with AAV-GFP or AAV-Tau^P301L^, as previously described [3]. Following immunohistochemistry, the slides were dehydrated and scanned using the Aperio Slide Scanner (Aperio, Vista, CA). The blinded quantification was performed using the ImageScope software (Aperio, Vista, CA). The analysis of annotated cortical and hippocampal regions was performed using a custom-designed color deconvolution algorithm, as previously described [10]. More specifically, the algorithm was used to detect and measure the optical density of the brown chromagen (tau) and the result was presented as a percentage of tau burden within an annotated region.

To measure the hippocampal area three sections for each animal were used. The quantification of hippocampal area was performed using the ImageScope software (Aperio, Vista, CA).

### Biochemical analyses

For biochemical analysis, sequential extraction of tau was performed, as previously described [3]. Briefly, 500μL and 275μL of TBS buffer (50 mM Tris, pH8.0, 274 mM NaCl, 5 mM KCl, 1 mM PMSF, and protease and phosphatase inhibitor cocktail) were added to cortex and hippocampus, respectively and homogenized using the manual pestles. For RNA extraction, 75μL of each homogenate was transferred to a new Eppendorf tube and stored in − 80 °C until further processing. The remaining homogenates were ultracentrifuged at 150,000×*g* for 15 min at 4 °C and the supernatant (S1 fraction) was transferred to a new Eppendorf tube. The pellet was homogenized in 3 × volume buffer in a high salt buffer (10 mM Tris, pH 7.4, 0.8 M NaCl, 10% sucrose, 1 mM EGTA, 1 mM PMSF and protease and phosphatase inhibitor cocktail) and ultracentrifuged at 150,000×*g* for 15 min at 4 °C. The pellet was discarded and the supernatant was transferred to a new Eppendorf tube and incubated with sarkosyl (final concentration of 1%) for 1 h at 37 °C. The incubation was followed by ultracentrifugation 150,000×*g* for 1 h at 4 °C. The supernatant was transferred to a new Eppendorf tube (S2 fraction) and the pellet was resuspended in the TE buffer (10 mM Tris, pH 8.0, 1 mM EDTA) (P3 fraction). All three fractions were stored in − 80 °C until further processing. Mouse CLU levels were measured using the Mouse Clusterin DuoSet ELISA (R&D Systems, DY2747), according to manufacturer’s instructions. To measure the total tau levels in the S1 and S2 fractions the MesoScale Discovery (MSD) was performed. Human tau specific E1 antibody (1:500) and biotinylated-HT7 antibody (1:250) were used as the capture and detection antibody, respectively. Tau levels were normalized to the total protein levels, measured by the Bicinchoninic Acid (BCA) Protein Assay kit (Thermo Scientific), according to the manufacturer’s instructions with a standard curve using BSA.

To examine total tau levels by immunoblotting, soluble fractions (S1) from cortical region of 6-month-old CLU WT-Tau^P301L^ and CLU KO-Tau^P301L^ mice were used. BCA assay was used to evaluate protein concentrations. Twenty micrograms from each sample was run on AnykD Criterion Protein Gels (BioRad) and transferred to PVDF membrane (Millipore). Membranes were blocked in 5% non-fat dry milk in TBS with 0.1% Triton-X-100 (TBST) for 1 h and incubated overnight at 4 °C in primary antibodies (anti-E1 1:2000 and anti-PHF-1 1:500) diluted in 5% milk in TBST. Membranes were then incubated in HRP-conjugated secondary antibodies (1:5000; Jackson ImmunoResearch) for 1 h at room temperature, and detected by ECL (Thermo Fisher Scientific, Rockford, IL). Bands were quantified by analyzing pixel density, and protein levels were normalized to the protein loading control (GAPDH).

### Tau filament assembly

Tau filament assembly was performed with 5 μM 4R0N isoform in assembly buffer (10 mMHEPES (pH7.4), 33 mM NaCl, 1 mM MgCl2, 1.5 mM EGTA, 60 μM EDTA, 5uM Thioflavin-T and 20ug/ml Heparin) with and without 4 μM recombinant CLU (R&D #2937-HS-050). Reactions were incubated at 37 °C and shaken in double orbital at 400 rpm on a BMG Fluostar platereader. Thioflavin-T fluorescence was measured every 30 min (450 ± 10 nm excitation, 480 ± 10 nm emission, bottom read). One silica bead (800 μm, Ops diagnostics) was added to each well in a 384 well plate format.

Following the polymerization reaction, samples were ultracentrifuged at 150,000×*g* for 40 min at 4 °C in a TLA110 rotor at 60,000 rpm to separate soluble and insoluble, aggregated tau. Pellets containing aggregated tau (insoluble fraction) were resuspended in 60 μL 1 × SDS-PAGE sample buffer (Invitrogen), and 10 μL 6 × SDS-PAGE sample buffer was added to 50uL of the supernatant (soluble fraction). Samples were separated by SDS-PAGE in Tris–glycine gels (Life Technologies), and transferred to PVDF membranes (Millipore). Membranes were blocked in 5% non-fat dry milk in TBS/0.1% Triton X-100, and incubated in primary antibody (E1 or biotinylated CLU). Membranes were then incubated in HRP-conjugated secondary antibodies for 1 h at RT and detected by ECL (Thermo Fisher Scientific, Rockford, IL).

Heparin-free tau assembly assay was also performed with 5 μM 4R0N, tau PHF seeds and increasing concentrations of recombinant CLU (0.25 μM–2 μM). To purify tau PHFs, 150 mg of cortical region from human AD cases was used for the sequential extraction, as described above. The final P3 pellets were resuspended in 75 μL of TE buffer and 2μL of P3 fraction was used in the tau assembly assay.

### Statistical analyses

Statistical significance of experiments involving two groups was assessed by Student’s *t* test. The analysis of three or more groups were performed by using one-way ANOVA. The behavioral analysis was performed by using two-way ANOVA with Fisher’s LSD test. Data present as mean ± S.E.M. For all statistical analyses GraphPad Prism software was used (GraphPad).

## Results

### CLU is present in tau aggregates and upregulated in human tauopathies

**Fig. 1:**
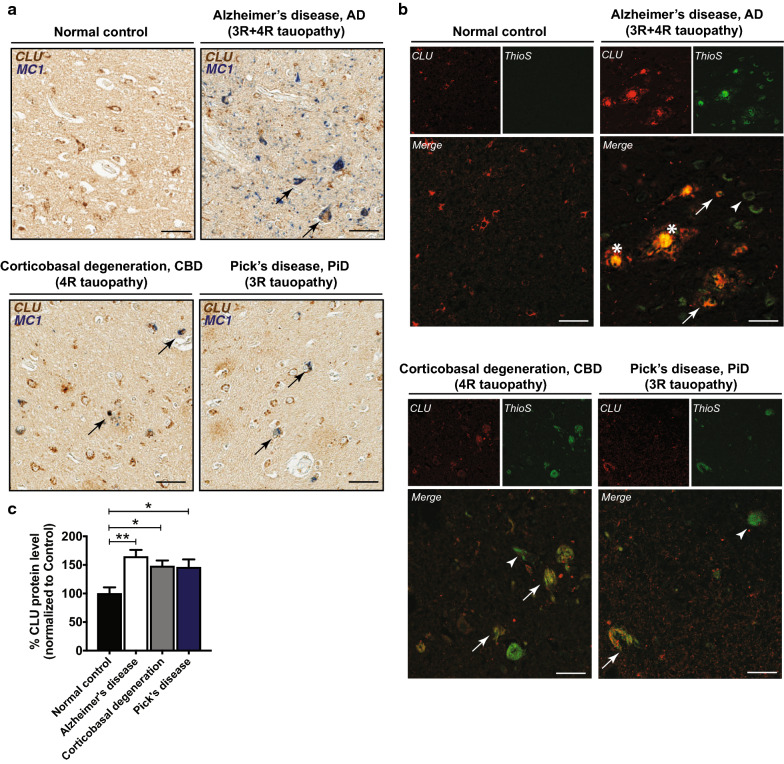
Clusterin co-localizes with tau deposits and is upregulated in human tauopathies. **a** Human brain tissues representing normal control, Alzheimer’s disease (AD), and the primary tauopathies Pick’s disease (PiD) and corticobasal degeneration (CBD). Co-localization of CLU (brown) with tau deposits, marked by MC-1 labeling (blue). Scale bar, 100 μm. **b** Arrows indicate co-localization of CLU (red) with mature tau tangles labeled by thioflavine-S staining (green). Arrowheads show tau tangles without CLU co-localization. Asterisks represent amyloid plaques. Scale bar, 100 μm. **c** Biochemical evaluation of the total CLU protein levels in the cortical region of human tauopathies. N = 10–15 cases/group. Data presented as mean ± S.E.M. and analyzed with one-way ANOVA with Tukey’s multiple comparison test, *p < 0.05, **p < 0.01

To determine the effect of CLU expression on tau deposition in the brain, we first evaluated the pattern of CLU localization in human primary and secondary tauopathies. Histological analyses of post-mortem brain tissues from individuals diagnosed with AD, CBD, and PiD (Additional file 1: Table S1) showed the association of CLU with MC-1-positive tau deposits (Fig. 1a). In addition, CLU co-localized with mature tau tangles, marked by thioflavine-S stain, suggesting the physiological interaction of CLU and tau in vivo (Fig. 1b). We further explored the link between CLU and tau pathology by assessing CLU protein levels in human tauopathies (Additional file 1: Table S2) with a quantitative enzyme-linked immunosorbent assay (ELISA). Consistent with previous reports, CLU was highly elevated in AD cases compared to normal controls, an effect partially attributed to the presence of abundant amyloid pathology in AD brains. Notably, we also found a significant increase in CLU levels in primary tauopathies with no amyloid pathology, including PiD and CBD compared to controls, indicating CLU upregulation in response to tau aggregation (Fig. 1c).

To further investigate the association of CLU with tau accumulation, we used a well-established mouse model of tauopathy [3] in which adeno-associated virus expressing human tau protein bearing the P301L mutation (AAV-Tau^P301L^) or green fluorescent protein as control (AAV-GFP) was delivered to CLU wild-type (WT) and CLU knock-out (KO) animals by intracerebroventricular injections at postnatal day 0. Consistent with our findings in human brains, CLU co-localized with tau aggregates (Additional file 1: Fig. S1a) and CLU levels were higher in CLU WT-Tau^P301L^ mice compared to control animals (Additional file 1: Fig. S1b).

### The absence of CLU leads to behavioral abnormalities

**Fig. 2:**
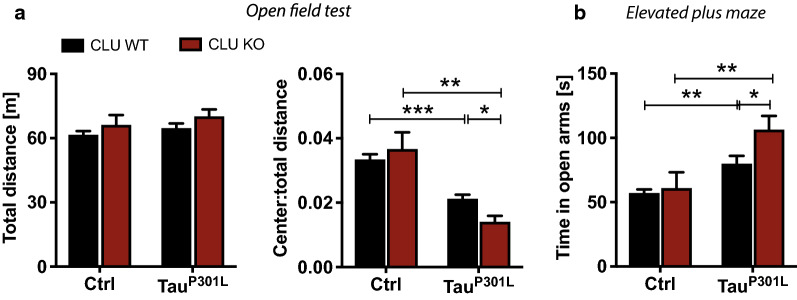
Loss of Clusterin leads to behavioral abnormalities. **a**-**b** Behavioral and cognitive abilities were assessed using **a** the open field test (OFA) and **b** elevated plus maze (EPM) in CLU WT-GFP (N = 49), CLU KO-GFP (N = 12), CLU WT-Tau^P301L^ (N = 65), and CLU KO-Tau^P301L^ (N = 23). OFA was used to test anxiety-related behavior, evaluated by time spent in the center compared to total time travelled. EPM measured the time spent in the open arms during the test which is a reflection of exploratory behavior. Data present as mean ± S.E.M. and analyzed with two-way ANOVA with Fisher’s LSD test, *p < 0.05, **p < 0.01, ***p < 0.001

Given that cognitive and behavioral impairment correlates with tau accumulation in AD patients and is also present in other tauopathies [1, 11], we assessed the effect of differential CLU expression on anxiety-like behavior as well as learning and memory functions. We did not observe significant differences between CLU genotypes in the total distance traveled (Fig. 2a). However, CLU KO-Tau^P301L^ mice had increased anxiety-like behavior, measured by the time spent in a center of the open field arena, compared to CLU WT-Tau^P301L^ (Fig. 2a). In addition, CLU KO-Tau^P301L^ mice spent significantly more time in the open arms of the elevated plus maze (EPM) test compared to CLU WT-Tau^P301L^ mice, an indication of aberrant exploratory behavior (Fig. 2b). CLU loss did not influence associative learning and memory assessed by the contextual and cued fear conditioning (CFC) tests (Additional file 1: Fig. S2). Overall, these data suggest a significant effect of CLU on anxiety-like behavior in the context of tau pathology.

### CLU loss exacerbates tau pathology in a mouse model of tauopathy

**Fig. 3:**
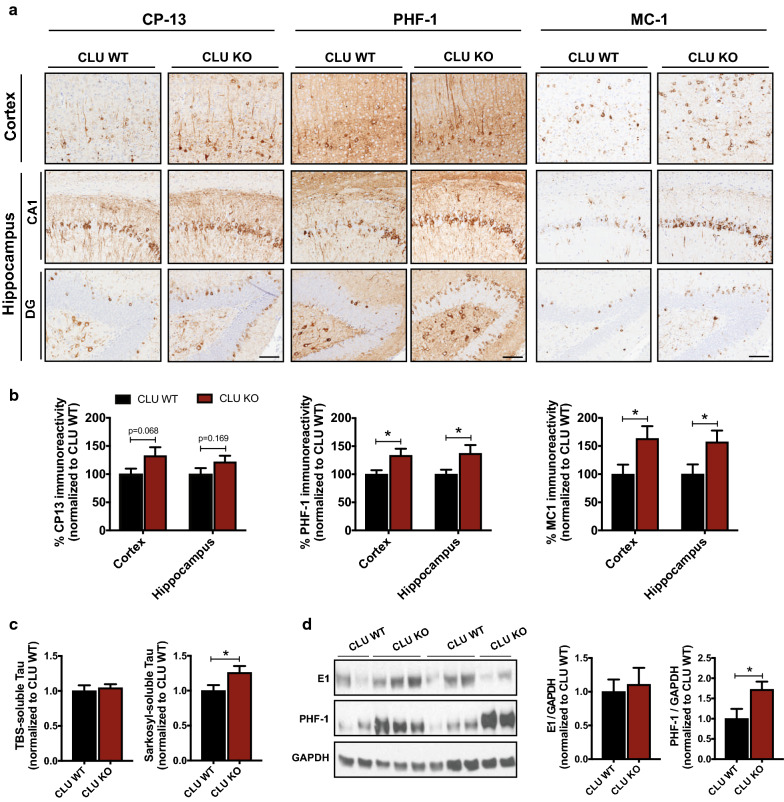
Clusterin reduces the severity of tau pathology. **a** Representative images from the cortical and hippocampal regions of 6-month-old CLU WT-Tau^P301L^ and CLU KO-Tau^P301L^ showing tau pathology. Tau hyperphosphorylation at serine 202, serines 396/404, and tau conformational change were detected by using CP-13, PHF-1, and MC-1, respectively. Scale bar, 100 μm. **b** Quantitative analysis of tau accumulation in cortex and hippocampus of CLU WT-Tau^P301L^ and CLU KO-Tau^P301L^ mice. For the CP-13, PHF-1 and MC-1 analyses n = 23–28 mice/group were used. Data presented as mean ± S.E.M. and analyzed with Student’s t test, *p < 0.05. **c** Biochemical evaluation of the total tau levels in the TBS-soluble (S1) and sarkosyl-soluble (S2) fractions. N = 22–25 mice/group. Data present as mean ± S.E.M. and analyzed with Student’s t test, *p < 0.05. **d** The levels of total and hyperphosphorylated tau were assessed by the immunoblotting analysis. N = 12 mice/group. Data present as mean ± S.E.M. and analyzed with Student’s t test, *p < 0.05

Given that tau is abnormally hyperphosphorylated in AD and other tauopathies [23], we next evaluated the pattern of phosphorylation at different epitopes in cortex and hippocampus of CLU WT-Tau^P301L^ and CLU KO-Tau^P301L^ animals. CLU KO-Tau^P301L^ mice had a modest increase in tau phosphorylation at serine 202 (pS202) detected by CP-13 immunolabeling in cortex (*p* = 0.068) and hippocampus (*p* = 0.169) compared to CLU WT-Tau^P301L^ animals (Fig. 3a, b). Since CP-13 detects an early stage of pathology, we also examined the degree of phosphorylation at the pS396 and pS404 sites that are thought to represent more mature tau deposits. Notably, the immunolabeling with PHF-1 showed significantly stronger immunoreactivity in the cortical (*p < 0.05) and hippocampal (*p < 0.05) regions of CLU KO-Tau^P301L^ mice compared to CLU WT-Tau^P301L^ animals (Fig. 3a, b). To further characterize the effect of CLU on tau pathology, we examined the abnormal conformational change of tau in these mice using MC-1 immunolabeling. Similar to the effects on PHF-1, CLU KO-Tau^P301L^ mice exhibited significantly higher MC-1 positive immunoreactivity in both cortex (*p < 0.05) and hippocampus (*p < 0.05) compared to CLU WT-Tau^P301L^ animals (Fig. 3a, b and Additional file 1: Fig. S3). Consistent with this, Gallyas silver stain, detecting mature tau fibrils, was more abundant in cortex of CLU KO-Tau^P301L^ mice (*p < 0.05); although, there was no significant difference in the Gallyas-positive deposits in the hippocampus between CLU WT-Tau^P301L^ and CLU KO-Tau^P301L^ mice (Additional file 1: Fig. S4a, b). In addition, mice lacking CLU exhibited a significantly smaller area of the hippocampus compared to control animals, suggesting that augmented tau pathology in these animals was accompanied by hippocampal atrophy (Additional file 1: Fig. S4c).

The effect of CLU expression on tau pathology led us to evaluate the impact of CLU on tau solubility by assessing tau levels biochemically. To this end, we isolated different tau species from cortical tissue and evaluated total tau levels in TBS-soluble and sarkosyl-soluble fractions, using a highly sensitive meso-scale discovery (MSD) assay. As expected, tau levels in the soluble fraction, reflecting less aggregated forms of tau, were not significantly different between CLU genotypes (Fig. 3c). However, we observed significantly higher levels of oligomeric tau in the sarkosyl-soluble fraction in CLU KO-Tau^P301L^ mice (*p < 0.05) compared to CLU WT-Tau^P301L^ animals (Fig. 3c). Immunobloting further showed a marked increase in the levels of phosphorylated tau (*p < 0.05) in cortex of CLU KO-Tau^P301L^ mice compared to control CLU WT-Tau^P301L^ animals, with no differences in the total tau levels observed between two groups (Fig. 3d). Taken together, these results indicate that the presence of CLU had a significant effect on tau pathology, particularly the more severe or mature forms of tau.

Previous studies have shown abundant gliosis associated with tau accumulation in the brain [22]. Thus, we performed a histological examination of astrogliosis using glial fibrillary acidic protein (GFAP) as a marker for reactive astrocytes. As expected, we observed marked astrogliosis in the cortex and hippocampus of mice injected with AAV-Tau^P301L^ (****p < 0.0001) compared to animals injected with AAV-GFP (Additional file 1: Fig. S5). Additionally, CLU genotype further influenced astrogliosis, with CLU KO-Tau^P301L^ mice having higher levels of astrogliosis in cortex (*p* = 0.055), but not hippocampus (*p* = 0.58) compared to CLU WT-Tau^P301L^ animals (Additional file 1: Fig. S5). We also observed a significant increase in microgliosis associated with tau deposits (**p < 0.01), but we did not find differences between the CLU genotypes (Additional file 1: Fig. S5).

### CLU significantly inhibits tau fibril formation

**Fig. 4:**
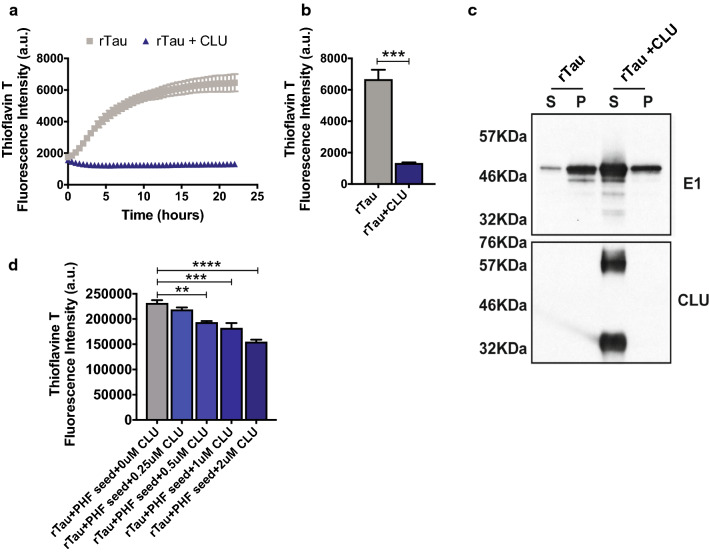
CLU binds to tau and prevents tau aggregation. **a** *In vitro* tau fibrilization assay was performed with the 4R0N tau isoform in the presence or absence of recombinant human CLU and thioflavine T intensity was monitored during 24-h time course. **b** Quantification of thioflavine T signal at the 24-h time point. Four replicates were used. Data present as mean ± S.E.M. and analyzed by Student’s t test ***p < 0.001. **c** Western blot analysis of soluble (supernatant, s) and insoluble (pellet, p) fractions following tau fibril assembly in the presence or absence of exogenous CLU. **d** Tau assembly assay performed with 4R0N recombinant tau, tau PHFs from AD brains, and increasing concentrations of recombinant CLU. Four replicates were used. Data present as mean ± S.E.M. and analyzed by one-way ANOVA, **p < 0.01, ***p < 0.001, ****p < 0.0001

To investigate a possible mechanism by which CLU may impact severity and progression of tau pathology, we evaluated the ability of CLU to influence tau fibrillization in a cell-free system. To this end, we incubated recombinant human tau protein (4R0N) with recombinant human CLU and assessed the effects over the course of 24 h. Strikingly, we observed that the presence of CLU significantly prevented the assembly and aggregation of tau (***p < 0.001) in this thioflavin T assay (Fig. 4a, b). Following tau fibrillization, we assessed CLU and tau levels in the remaining soluble and insoluble fractions using Western blot analysis. While the absence of CLU in the preparations led to the robust aggregation of tau in the insoluble fraction, the presence of CLU promoted tau solubility as detected by a higher amount of CLU and tau retained in the soluble fraction with only small amount of tau present as insoluble aggregates (Fig. 4c).

Importantly, a similar effect was observed when recombinant tau was incubated with tau paired helical filaments (PHFs) purified from AD brains, and with increasing CLU concentrations. The presence of CLU led to a significant reduction of the tau assembly measured by thioflavine T fluorescence (Fig. 4d).

## Discussion

In the present study, we examined the functional relationship between CLU and tau using the AAV-based approach to deliver human tau bearing the P301L mutation into CLU WT and CLU KO animals. We found that loss of CLU was associated with the aggravated accumulation of pathological tau and behavioral abnormalities in our mouse model of tauopathy. We also found that CLU co-localized with tau deposits in human tauopathy cases and that direct interaction of CLU and tau in a cell-free system led to a dramatic reduction of the tau filament formation.

CLU is upregulated in AD cases and numerous genetic studies have found a strong association of the protective *T* allele of the rs11165000 SNP in the *CLU* gene with higher CLU levels in AD patients [9, 13, 20], suggesting that the increased CLU expression may be beneficial. In addition, the link between elevated CLU and tau pathology has been recently reported in AD patients [21]. Consistent with these findings, we observed highly upregulated levels of CLU in AD patients compared to control individuals. Importantly, our study is the first to show a significant increase in CLU levels in primary tauopathies compared to controls, suggesting that CLU upregulation occurs not only in the presence of amyloid plaques but also tau pathology. As previously shown [6], we found an extensive CLU co-localization with amyloid deposits in AD brains. Moreover, CLU immunoreactivity was present in many NFT-bearing neurons in both primary and secondary tauopathies suggesting tau-specific changes in CLU expression.

Importantly, our study also showed that CLU significantly influenced the formation of insoluble tau filaments in vitro, thus elucidating a possible mechanism by which CLU may modulate tau aggregation. However, it remains unclear where the in vivo interaction between CLU and tau may occur. Although tau predominantly exists as an intracellular protein, recent studies have suggested that tau can be also released from neurons and undergo cell-to-cell transmission [23]. Given that CLU is mainly a secreted protein [5], the potential binding to tau may occur in the extracellular space, possibly influencing tau spreading. Previous studies have also suggested the existence of intracellular forms of CLU that would emerge from alternative splicing, translation-initiation events, or failed translocation. Leskov and colleagues have reported the expression of the nuclear form of CLU that locates to the nucleus in response to cellular stress [14]. Other intracellular CLU forms have also been proposed, including partially-glycosylated form of secreted CLU translocated to the cytosol or mitochondrial membrane, therefore escaping the secretory pathway [18]. A recent report has shown the interaction of intracellular CLU with the wild-type and P301L mutant tau protein in vitro, suggesting that CLU may also regulate tau accumulation by its binding inside neurons [26].

Two recent studies have centered on defining the role of another abundantly expressed apolipoprotein in the brain and a major AD risk gene, apolipoprotein E (ApoE), in tau pathology [22, 25]. Shi and colleagues have shown that *APOE ε4* induced a more severe disease phenotype with augmented neurodegeneration and neuroinflammation compared to *APOE ε3* and *APOE ε2* genotypes in P301S transgenic mice [22]. Another study led by Zhao and colleagues has reported a strong link between *APOE ε2* and increased pathological changes of tau in the brains of mice injected with AAV-Tau^P301L^ [25]. Moreover, the authors have found a significant association between the *APOE ε2/ε2* genotype and increased risk of progressive supranuclear palsy [25]. Although these two seminal reports have demonstrated the associations of exacerbated tau pathology with different APOE isoforms, an effect possibly attributable to different model systems, nonetheless they provided new insights into APOE function in AD. It remains to be elucidated whether CLU and apoE impact the tau accumulation through the same mechanism in human tauopathies.

Although the effect of CLU on amyloid pathology has been well established, our findings suggest that CLU also plays a significant role in modulating tau pathology, thus providing new insights into CLU contribution to AD pathophysiology. Future studies into detailed mechanisms underlying the relationship of CLU with tau, perhaps in concert with apoE, may advance our understanding of AD and other tauopathies.

## Supplementary information

**Additional file 1.** **Table S1** Neuropathological information of samples used in the histological study. **Table S2** Demographics and neuropathological characteristics of human subjects. **Figure S1** CLU is present in tau aggregates and is upregulated in AAV-Tau^P301L^ animals. **a** Brain tissues of 6-month-old wild-type (WT) mice injected with AAV-GFP (Ctrl) and AAV-Tau^P301L^. Arrows show CLU co-localization with tau deposits. Arrowheads represent tau tangles without CLU immunoreactivity. Scale bar, 100 μm. **b** Quantification of CLU protein levels in cortex of WT mice injected with AAV-GFP (Ctrl) and AAV-Tau^P301^. N = 15–16 mice/group. Data presented as mean ± S.E.M. and analyzed with Student’s t test, *p < 0.05. **Figure S2** CLU loss does not impact associative learning in the AAV-Tau^P301L^ mouse model. **a** Contextual and **b** cued fear conditioning (CFC) test was performed to evaluate hippocampal-dependent **a** and amygdala-dependent **b** learning and memory. CLU WT-GFP (N = 49), CLU KO-GFP (N = 12), CLU WT-Tau^P301L^ (N = 65), and CLU KO-Tau^P301L^ (N = 23). Data presented as mean ± S.E.M. and analyzed with two-way ANOVA with Fisher’s LSD test, *p < 0.05. **Figure S3** CLU influences accumulation of MC-1-positive tau deposits. **a** MC-1 immunoreactivity in cortex and hippocampus of CLU WT-Tau^P301L^ and CLU KO-Tau^P301L^ mice. Scale bar, 400μm. **Figure S4** Loss of CLU is associated with increased accumulation of mature tau fibrils. **a** Gallyas silver stain was used to detect mature tau fibrils in cortex and hippocampus of 6-month-old CLU WT and CLU KO mice, injected with AAV-Tau^P301L^ virus. Scale bar, 100 μm. **b** Quantification of Gallyas stain in cortex and hippocampus of CLU WT-Tau^P301L^ and CLU KO-Tau^P301L^ mice. N = 17–21 mice/group. Data presented as mean ± S.E.M. and analyzed with Student’s *t* test, *p < 0.05. **c** Hippocampal area was measured in CLU WT-Tau^P301L^ and CLU KO-Tau^P301L^ mice. N = 16–20 mice/group. For each animal three sections were analyzed. Data presented as mean ± S.E.M. and analyzed with Student’s *t* test, *p < 0.05. **Figure S5** CLU loss has a modest influence on tau-associated astrogliosis but not microgliosis**. a** Representative images showing astrogliosis in cortex and hippocampus of 6-month-old CLU WT and CLU KO mice injected with AAV-GFP or AAV-Tau^P301L^. Scale bar, 100 μm. **b** Quantitative analysis of GFAP immunoreactivity in cortex and hippocampus. AAV-GFP N = 6–8 mice/group, AAV- Tau^P301L^ N = 22–24 mice/group. Data present as mean ± S.E.M. and analyzed with two-way ANOVA followed by Tukey correction for multiple comparisons, *p < 0.05, **p < 0.01. **c** Microgliosis was evaluated by IBA1 immunoreactivity in cortex and hippocampus of CLU WT and CLU KO mice injected with AAV-GFP or AAV-Tau^P301L^. Scale bar, 100 μm. **d** Quantification of IBA1 immunoreactivity in cortex and hippocampus. AAV-GFP n = 6–8 mice/group, AAV- Tau^P301L^ N = 22–24 mice/group. Data present as mean ± S.E.M. and analyzed with two-way ANOVA followed by Tukey correction for multiple comparisons.

## Data Availability

All data used and analyzed for the current study are available from the corresponding author on reasonable request.

## Abbreviations

AAV: Adeno-associated viral vectors
AD: Alzheimer’s disease
APOE: Apolipoprotein E
CBD: Corticobasal degeneration
CLU: Clusterin
ELISA: Enzyme-linked immunosorbent assay
GFAP: Glial fibrillary acidic protein
GFP: Green fluorescent protein
GWAS: Genome-wide association studies
IBA1: Ionized calcium binding adaptor molecule 1
MSD: Meso Scale Discovery
PiD: Pick’s disease
PSP: Progressive supranuclear palsy
SNP: Single nucleotide polymorphism
TBS: Tris-buffered saline
