# Supplementary material for: Clusterin ameliorates tau pathology in vivo by inhibiting fibril formation
Source: Acta Neuropathol Commun. 2020 Dec 1;8:210. doi: 10.1186/s40478-020-01079-1 (PMC7708249; doi:10.1186/s40478-020-01079-1)
Supplement: Supplementary file 1 — Additional file 1. Table S1 Neuropathological information of samples used in the histological study. Table S2 Demographics and neuropathological characteristics of human subjects. Figure S1 CLU is present in tau aggregates and is upregulated in AAV-TauP301L animals. a Brain tissues of 6-month-old wild-type (WT) mice injected with AAV-GFP (Ctrl) and AAV-TauP301L. Arrows show CLU co-localization with tau deposits. Arrowheads represent tau tangles without CLU immunoreactivity. Scale bar, 100 μm. b Quantification of CLU protein levels in cortex of WT mice injected with AAV-GFP (Ctrl) and AAV-TauP301. N = 15–16 mice/group. Data presented as mean ± S.E.M. and analyzed with Student’s t test, *p < 0.05. Figure S2 CLU loss does not impact associative learning in the AAV-TauP301L mouse model. a Contextual and b cued fear conditioning (CFC) test was performed to evaluate hippocampal-dependent a and amygdala-dependent b learning and memory. CLU WT-GFP (N = 49), CLU KO-GFP (N = 12), CLU WT-TauP301L (N = 65), and CLU KO-TauP301L (N = 23). Data presented as mean ± S.E.M. and analyzed with two-way ANOVA with Fisher’s LSD test, *p < 0.05. Figure S3 CLU influences accumulation of MC-1-positive tau deposits. a MC-1 immunoreactivity in cortex and hippocampus of CLU WT-TauP301L and CLU KO-TauP301L mice. Scale bar, 400μm. Figure S4 Loss of CLU is associated with increased accumulation of mature tau fibrils. a Gallyas silver stain was used to detect mature tau fibrils in cortex and hippocampus of 6-month-old CLU WT and CLU KO mice, injected with AAV-TauP301L virus. Scale bar, 100 μm. b Quantification of Gallyas stain in cortex and hippocampus of CLU WT-TauP301L and CLU KO-TauP301L mice. N = 17–21 mice/group. Data presented as mean ± S.E.M. and analyzed with Student’s t test, *p < 0.05. c Hippocampal area was measured in CLU WT-TauP301L and CLU KO-TauP301L mice. N = 16–20 mice/group. For each animal three sections were analyzed. Data presented as mean ± S.E.M. and analyzed with Student’s t [file 40478_2020_1079_MOESM1_ESM.pdf]

Neuropathological diagnosis	Normal Control	Alzheimer's disease	Corticobasal degeneration	Pick's disease
Braak tangle stage	1	5	3	2
Thal amyloid phase	0	5	0	0
Age	73	69	75	59

Neuropathological diagnosis	Normal Control (N=10)	Alzheimer's disease (N=10)	Corticobasal degeneration (N=15)	Pick's disease (N=12)
Median Braak tangle stage (range)	2 (1-3)	6 (6)	2 (0-4)	2 (0-3)
Median Thal amyloid phase (range)	0 (0-1)	5 (5)	0 (0-2)	0 (0-3)
Median age (range)	71.5 (60-78)	66 (56-75)	67 (58-77)	65.5 (53-84)
Female (%)	4/10 (40%)	6/10 (60%)	6/15 (40%)	5/12 (42%)

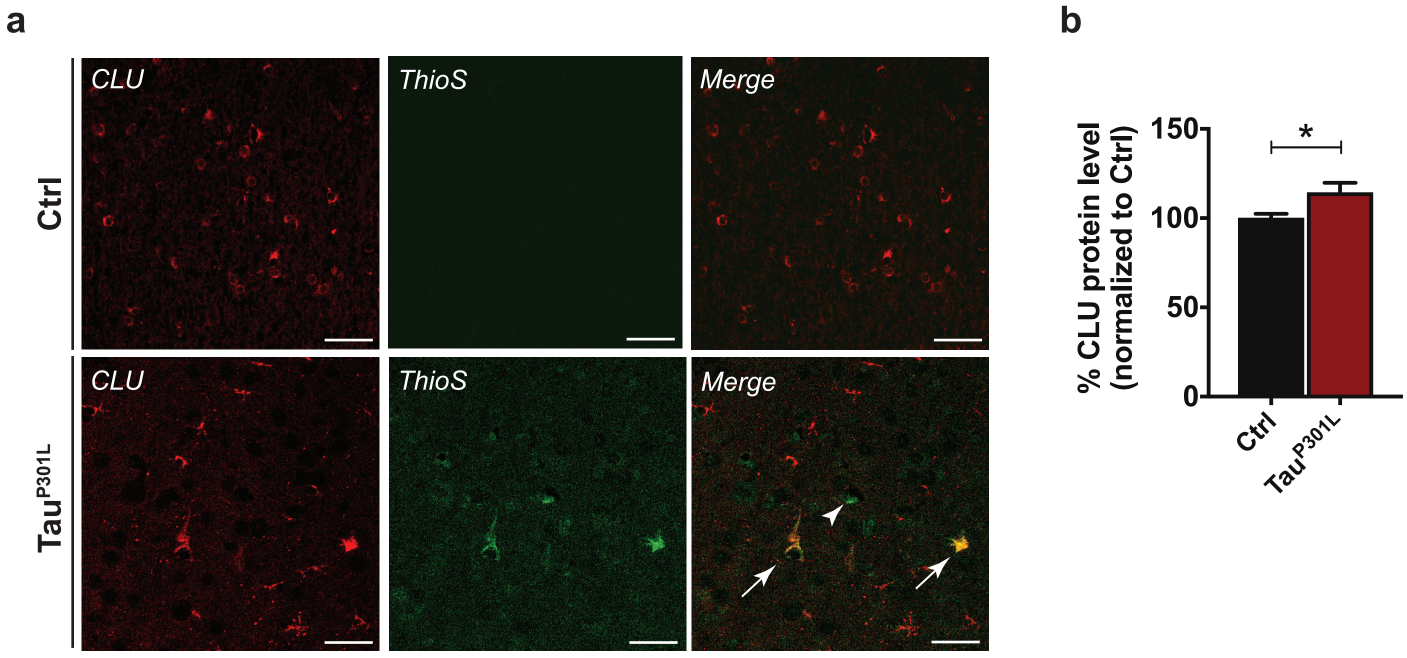

a
Ctrl
Tau^P301L
CLU
ThioS
Merge
b
% CLU protein level
(normalized to Ctrl)
150
100
50
0
*
Ctrl
Tau^P301L

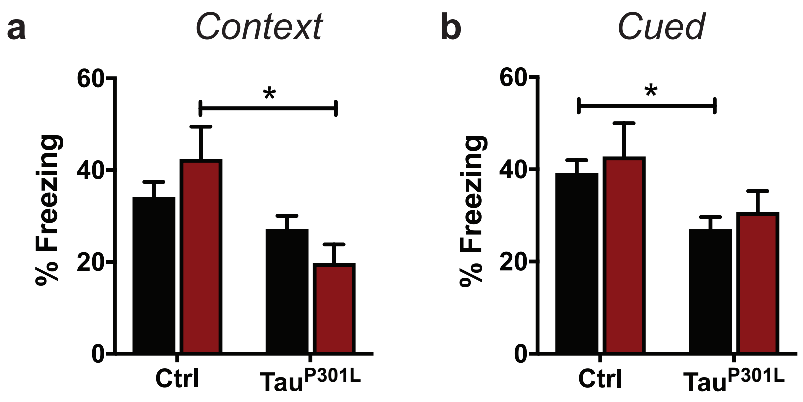

a
Context
b
Cued
60
40
20
0
% Freezing
*
Ctrl
TauP301L

a

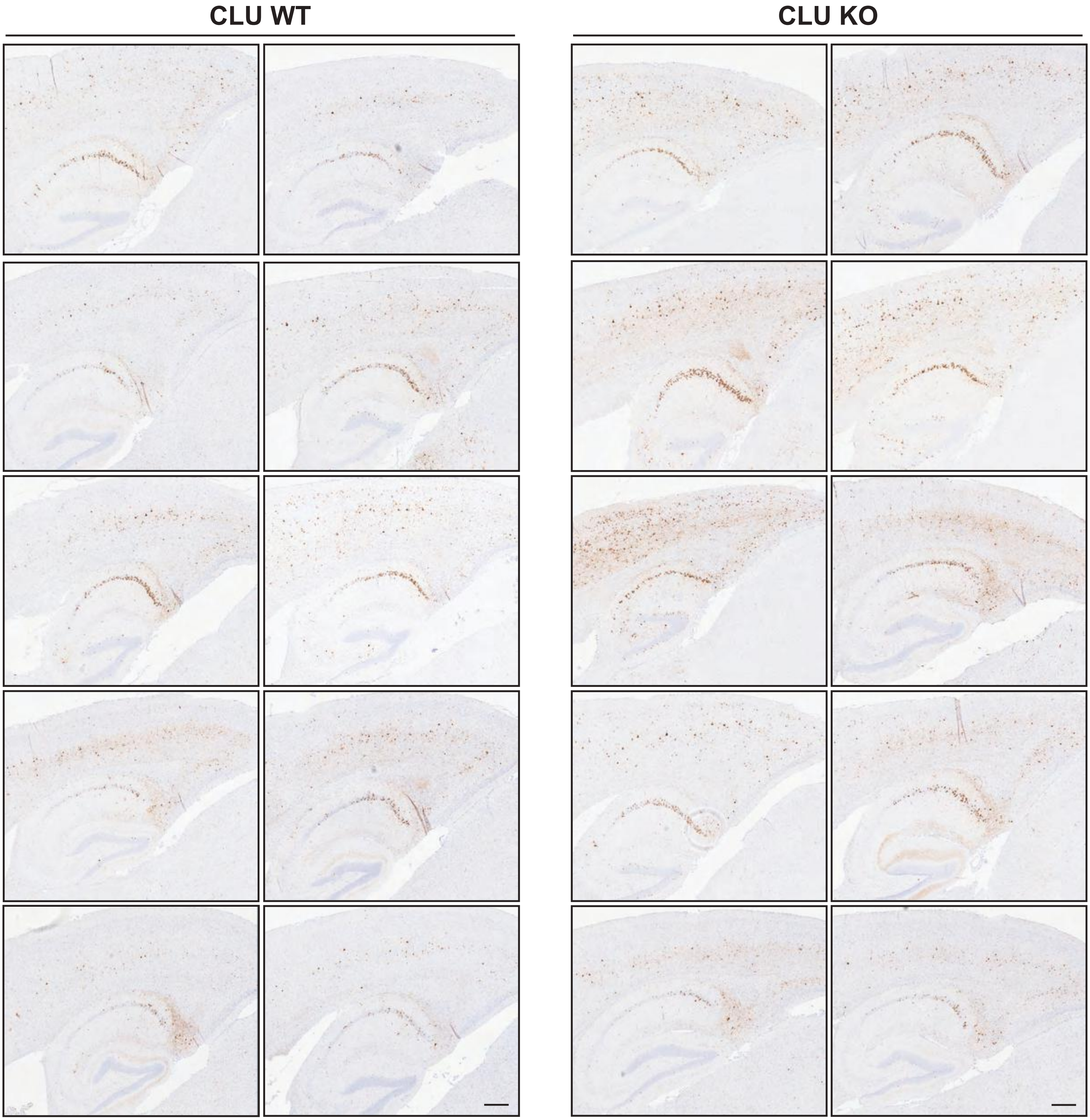

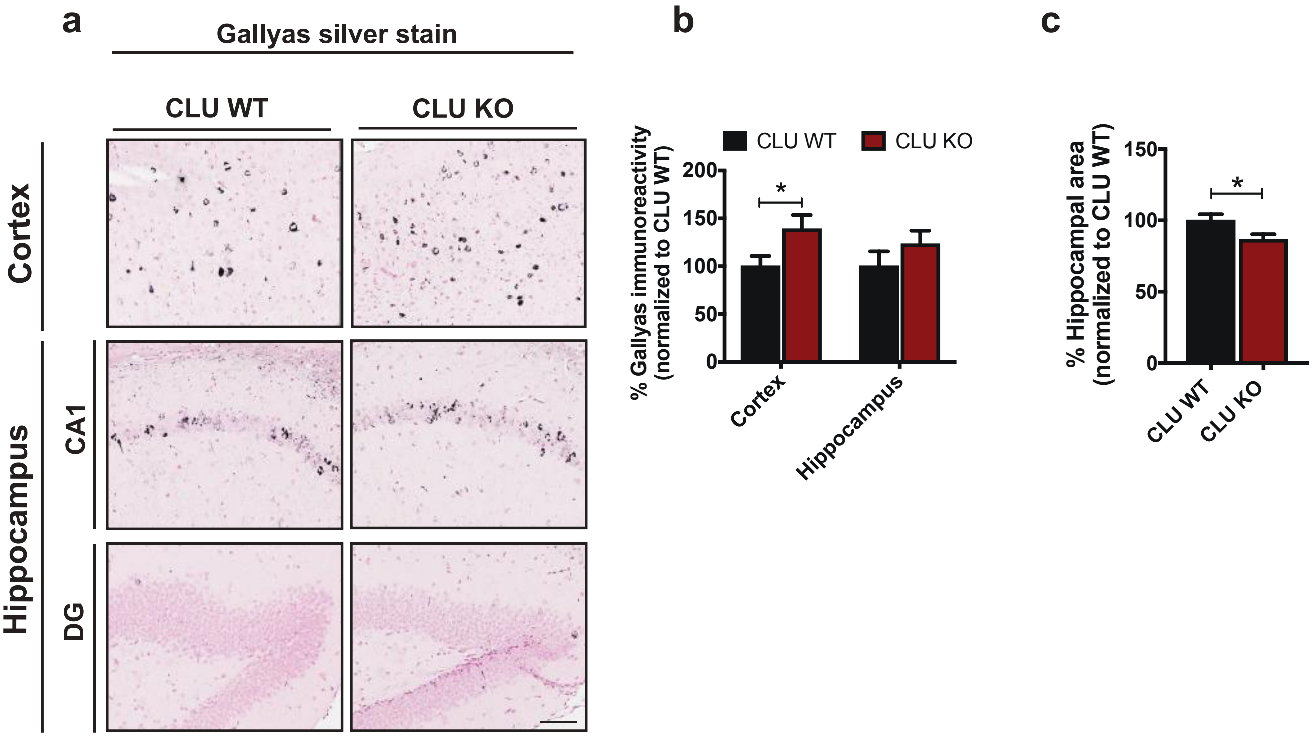

a
Gallyas silver stain
CLU WT
CLU KO
Cortex
Hippocampus
CA1
DG
b
CLU WT
CLU KO
% Gallyas immunoreactivity (normalized to CLU WT)
200
150
100
50
0
*
Cortex
Hippocampus
c
% Hippocampal area (normalized to CLU WT)
150
100
50
0
*
CLU WT
CLU KO

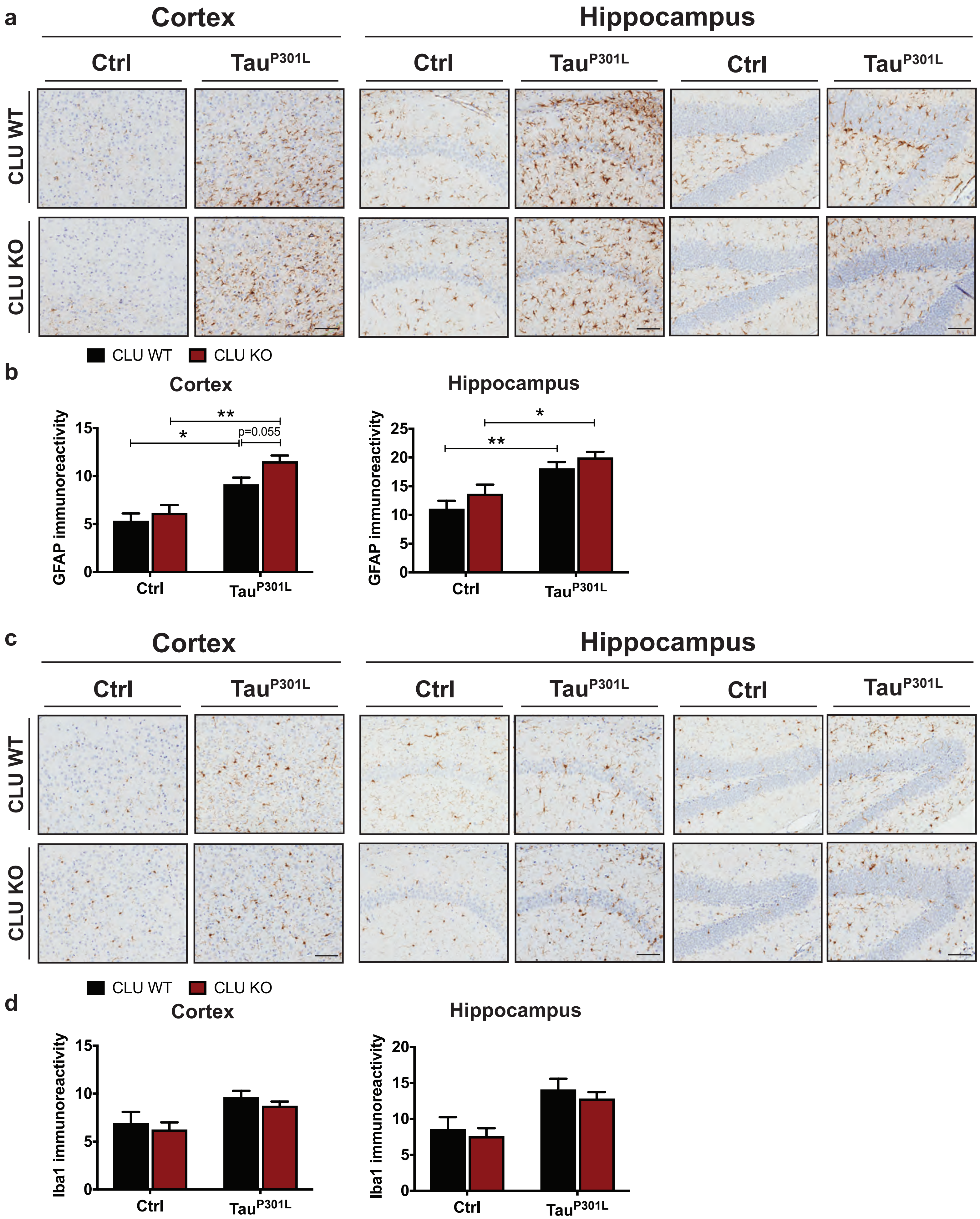

a
Cortex
Hippocampus
Ctrl
Tau^{P301L}
Ctrl
Tau^{P301L}
Ctrl
Tau^{P301L}
CLU WT
CLU KO
CLU WT
CLU KO
b
Cortex
Hippocampus
GFAP immunoreactivity
**
*
p=0.055
*
**
Ctrl
Tau^{P301L}
c
Cortex
Hippocampus
Ctrl
Tau^{P301L}
Ctrl
Tau^{P301L}
Ctrl
Tau^{P301L}
CLU WT
CLU KO
CLU WT
CLU KO
d
Cortex
Hippocampus
Iba1 immunoreactivity
Ctrl
Tau^{P301L}
